# siRNA-Finder (si-Fi) Software for RNAi-Target Design and Off-Target Prediction

**DOI:** 10.3389/fpls.2019.01023

**Published:** 2019-08-15

**Authors:** Stefanie Lück, Tino Kreszies, Marc Strickert, Patrick Schweizer, Markus Kuhlmann, Dimitar Douchkov

**Affiliations:** ^1^Leibniz Institute of Plant Genetics and Crop Plant Research, Seeland, Germany; ^2^Institute of Cellular and Molecular Botany, University of Bonn, Bonn, Germany; ^3^Physics II Institute, University of Giessen, Giessen, Germany

**Keywords:** si-Fi, RNA interface, off-target, RNAi design, RNAi efficiency prediction, posttranscriptional gene silencing

## Abstract

RNA interference (RNAi) is a technique used for transgene-mediated gene silencing based on the mechanism of posttranscriptional gene silencing (PTGS). PTGS is an ubiquitous basic biological phenomenon involved in the regulation of transcript abundance and plants’ immune response to viruses. PTGS also mediates genomic stability by silencing of retroelements. RNAi has become an important research tool for studying gene function by strong and selective suppression of target genes. Here, we present **si-Fi**, a software tool for design optimization of RNAi constructs necessary for specific target gene knock-down. It offers efficiency prediction of RNAi sequences and off-target search, required for the practical application of RNAi. **si-Fi** is an open-source (CC BY-SA license) desktop software that works in Microsoft Windows environment and can use custom sequence databases in standard FASTA format.

## Introduction

High-throughput gene silencing technologies were applied in several organisms to study gene function. Although the novel technique of CRISPR/Cas9-directed site-specific mutagenesis is attracting wide scientific interest, RNAi still offers advantages. RNAi is able to target any transcript regardless of ploidy, and it is not hampered by chromatin structure modifications (Uusi-Makela et al., 2018). Furthermore, the RNAi-mediated silencing is quantitative (knockdown) and can be directed to tissue or developmental specificity by the utilization of the respective promoter. As this application is based on sequence similarity of the silencing trigger to the respective targets, it allows silencing of several gene family members with a unique construct.

RNAi can be used for stable as well as for transient transgene-mediated gene silencing based on the mechanism of posttranscriptional gene silencing (PTGS) (Unniyampurath et al., 2016). PTGS is an essential part of plant immune response to viruses (Muhammad et al., 2019) and required for genomic stability by silencing of retroelements (Almeida and Allshire, 2005).

Small interfering RNAs (siRNAs) are the hallmark of posttranscriptional gene silencing (PTGS). These small RNAs, usually 21 nucleotides (nt) in length, mediate the sequence specificity and represent the active principle of successful gene silencing (Zamore et al., 2000; Elbashir et al., 2001). They are generated by a DICER-mediated cleavage of double-stranded RNA (dsRNA) into 19–25 base pair (bp) long double-stranded oligonucleotides with 2-nt 3’overhangs. A sequence asymmetry-sensing mechanism is selecting one strand to be incorporated into an ARGONAUT-containing complex named RISC (RNA-induced silencing complex) (Khvorova et al., 2003; Schwarz et al., 2003; Amarzguioui and Prydz, 2004; Reynolds et al., 2004; Ui-Tei et al., 2004; Frank et al., 2010; Walton et al., 2010; Noland et al., 2011). The mRNA targets are then found by nucleotide pairing between the antisense (guide) strand of the AGO-incorporated siRNA on the one side and the target mRNA on the other. This interaction is probably influenced—besides other factors—by the physical accessibility and presence of unpaired nucleotides at the RNA target site (Ameres et al., 2007). Once the guide siRNA chain of the silencing complex is paired to the target mRNA molecule, the latter will be hydrolyzed, and thus, the expression of the corresponding gene will be knocked down (Hammond et al., 2000; Bernstein et al., 2001).

RNAi has become widely used as a tool to modulate gene expression. A vast amount of experimental data has been accumulated, which allows design and validation of RNAi prediction models (McGinnis, 2010). Several publicly available software tools, mainly intended for their use in mammals, are described for the design of dsRNA silencing inducers (Naito and Ui-Tei, 2012; Schmidt et al., 2013). Also, software from commercial distributors is available [e.g., siRNA Wizard Software 3.1, InvivoGen (Lan et al., 2009) or BLOCK-iT™ RNAi Designer, Thermofisher (Chung et al., 2017)]. In mammals, long double-stranded RNA molecules are known to induce strong interferon reactions and cell death (Isaacs and Lindenmann, 1957). Therefore, the intention in animal and human research is to use short double-stranded oligonucleotides for triggering the silencing. This also influenced the development of experimental techniques and software tools. Due to the absence of the interferon response in plants, the use of long double-stranded RNA as a trigger of RNA is possible. However, data on the experimental validation of several different constructs used for silencing in plants are more limited.

In order to validate the presented **si-Fi** algorithm, we performed experiments to test the algorithm output. The experimental system is based on transient transformation protocols *via* particle bombardment of barley (*Hordeum vulgare* spp. *vulgare*) leaf segments. In our experimental approach, specific genes are targeted to improve the resistance against barley powdery mildew pathogen *Blumeria graminis* f.sp. *hordei (Bgh)*. Barley and *Bgh* represent a plant–pathogen system with a model character due to a large body of published phytopathological, genetic, physiological, and molecular data (Huckelhoven and Panstruga, 2011; Bindschedler et al., 2016). Cultivated barley is among the most important crop plants worldwide. Besides its agronomic importance, barley is an established model organism of the *Triticeae* tribe of grasses. It has a large amount of genetic and genomic resources, large *ex situ* germplasm collections, breeding knowledge, and a well-annotated genome (Mayer et al., 2012; de Carvalho et al., 2013; Milner et al., 2018). *Blumeria graminis* is an obligate biotrophic *Acomycota* fungus with extreme host specificity [split up into so-called *formae speciales* (f. sp.) e.g. *hordei* for barley]. The genome sequence information is available (Spanu et al., 2010). Fungal development is fast and synchronous with entirely epiphytic hyphal growth on leaf or stem surfaces. The prerequisite of a successful fungal infection is the generation of a haustorium. Haustoria (specialized nutrient-uptake cells) are localized inside the host epidermal layer. In our study, we scored the plant susceptibility by observing the first and most critical step in this interaction—the establishment of the first fungal haustorium in the host cell.

In barley, one of the best studied resistance genes for powdery mildew is *HvMlo* (HORVU4Hr1G082710). *HvMlo* was identified as a functional genetic component in the resistance mediating Mildew locus O (Jorgensen, 1992) and encodes a seven-transmembrane domain protein (Buschges et al., 1997), representing a susceptibility factor of the basal plant defense reactions (Appiano et al., 2015). The 4,388-bp long gene contains a coding region of 3,145 bp with 12 exons (**Supplementary Figure S1**). The annotated 5’UTR consists of 450 bases and the 3’UTR of 1,100 bases. Functional repression of the gene by silencing or complete knockout confers strong and broad-spectrum resistance to *Bgh* (Acevedo-Garcia et al., 2014). As the response is restricted to a limited number of transiently transformed epidermal cells, the molecular silencing effect, i.e., reduction of *HvMlo* mRNA abundance, is difficult to detect. Therefore, *Bgh* resistance represents an easy readout phenotype for efficient silencing of the *HvMlo* gene (Douchkov et al., 2005).

The *HvMloH1* (HORVU4Hr1G082760, GenBank Acc. Nr. Z95496.1) shows high sequence similarity to the *HvMlo* gene (about 85% identity) and potentially originates from a gene duplication. It can therefore be considered as potential off-target of the *HvMlo* gene. The functional information on this gene is very limited. The gene seems to be expressed to a very low level, only detectable in senescing leaves (Mascher et al., 2017).

Here, we present an approach to address the questions of RNAi specificity and design optimization by means of computer-aided prediction followed by experimental validation based on RNAi of the *HvMlo* gene and host resistance as indirect readout of RNAi efficiency.

## Materials and Methods

### DNA Constructs and Cloning

Synthetic DNA sequences (500 bp: 1,275 to 1,774 nucleotides from the coding region of NCBI GenBank Acc. Nr. Z83834.1) were ordered from GenScript (Piscataway, NJ, USA) as pUC57 cloned inserts. For the design of the “molecular clock” constructs (see **Table 1**), the *HvMlo* sequence (HORVU4Hr1G082710, NCBI GenBank Acc. Nr. AK248332.1) was named “0MY”. With increased number of randomized nucleotides on random position, the constructs are named from 0MY (unmodified) to 50MY (complete random) as indicated in **Table 1**. For the accessibility approach, 100-bp nonoverlapping fragments of 0MY, designated according to their position “1–100W (window),” were inserted into the same position of the fully randomized 50MY sequence. The *HvMloH1* sequence was derived from the *HvMloH1* gene (HORVU4Hr1G082760, GenBank Acc. Nr. Z95496.1) as 500-bp sequence homologous to the 0MY construct. All synthetic sequences used are available at Douchkov et al. (2018). The synthetic DNA fragments were excised as SalI/XbaI fragments and cloned into pIPKTA38 vector (GenBank Acc. Nr. EF622216.1) and transferred as two oppositely oriented fragments into the RNAi vector pIPKTA30N (GenBank Acc. Nr. EF622218.1) by a Gateway™ LR reaction as described before (Douchkov et al., 2005).

**Table 1:** Properties of the synthetic DNA sequences based on the “molecular clock” model for RNAi trigger lengths of 500 bp.

Sequence name	Percent of randomized nucleotides	Observed percent identity to the target	Total siRNA hits in Mlo
0MY	0	100.0	480
1MY	2	98.0	304
2MY	4	96.0	205
3MY	6	94.4	149
4MY	8	92.2	101
5MY	10	90.2	59
10MY	20	89.4	0
15MY	30	74.8	0
20MY	40	67.6	0
25MY	50	60.4	0
30MY	60	66.0	0
35MY	70	48.6	0
40MY	80	46.8	0
45MY	90	42.2	0
50MY	100	34.2	0

### RNAi Constructs, Plants and Fungi

RNAi constructs for transient-induced gene silencing (TIGS) were transferred into barley leaf epidermal cells by particle bombardment as described (Douchkov et al., 2005). Agar-mounted detached leaf segments were inoculated 3 days after the bombardment with Swiss *Bgh* field isolate CH4.8 at a density of 150–200 conidia per square millimeter. Transformed GUS-stained epidermal cells as well as haustoria-containing transformed (susceptible) cells were counted 48 h after inoculation, and TIGS effects were statistically analyzed according to Douchkov et al. (2014).

### Software Libraries and Tools

**si-Fi** has been developed as a Python (v. 2.7) graphical user interface (GUI) for Microsoft Windows™-based systems. The following software libraries and bindings were used: Numpy (van der Walt et al., 2011); Qt 4.6 (Riverbank Computing Ltd, UK); PyQt4 Python bindings, Matplotlib (Hunter, 2007); Biopython (Cock et al., 2009); Seaborn (Waskom, 2016); Python Imaging Library (Lundh, 2009); BOWTIE short sequence aligner (Langmead et al., 2009); and Rnaplfold of Vienna RNA Package 2.0 (Lorenz et al., 2011). The **si-Fi** source code is available at Lück et al. (2017b). The MS Windows executable installation file of the version used in this article is available at Lück et al. (2017a). The **si-Fi** software is intended for off-line work and gives the advantage that non-public available genome information can be utilized for the target region identification. Furthermore, custom databases with specific sequences (e.g., gene families, synthetic sequences, etc.) can be used for target prediction. As some online tools are not supported over time, we make our software freely available to the user after downloading.

## Implementation

### Off-Target Searching and Prediction

The off-target searching pipeline starts with splitting a long RNAi trigger sequence (the complement to the target sequence of the corresponding RNA) into all possible x-mers, where x is the selected length of the siRNA. The derived x-mers are then used to probe an mRNA or cDNA sequence database for matching sequences. The exact algorithm for sequence similarity search BOWTIE (Langmead et al., 2009) was chosen because of its accuracy, speed, and memory efficiency. Although the primary goal of the software is to be used for sequence assembly in genome sequencing projects, it is also well applicable as target-finding tool to match and map small RNAs to their respective targets.

### siRNA Asymmetry and Strand Selection Parameters

**Figure 1:**
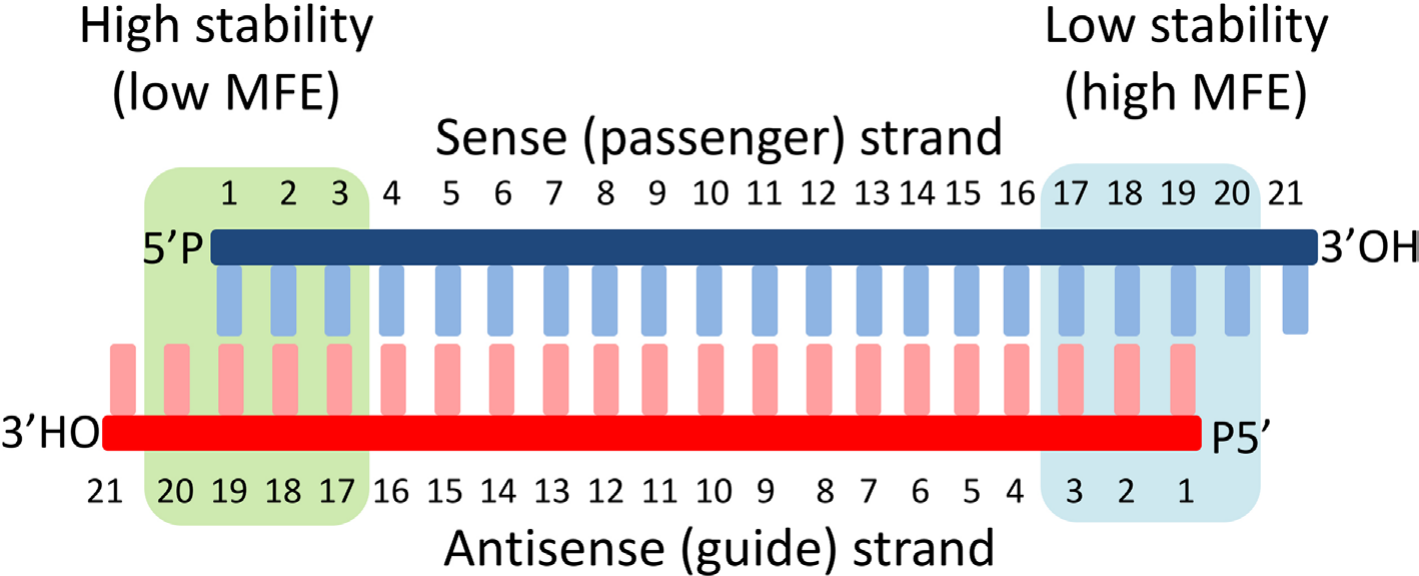
Basic structure of siRNAs and the principles of strand selection. The siRNA is central to the RNA interference. They are usually produced by digestion of long double-stranded RNA fragments to small double-stranded fragments with specific lengths (∼20 nucleotides) by RNAse III enzymes of Dicer type. Each fragment is then unwound by helicases into two single RNA strands, where only one of the strands (guide strand) is incorporated by an argonaute protein (AGO) into a RNA-induced silencing complex (RISC) and the other (passenger strand) is discarded. The discrimination mechanism for strand selection involves sensing of the thermodynamic stability of the 5’-ends of both strands and selection of those with lower 5’-end stability as a guide. In our approach, we calculate the minimum free energy (MFE) of both ends of the duplex, and the strand with the lower stability (high MFE) at its 5’-end is selected as a guide strand.

Since the guide strand is selected from the double-stranded siRNA, it is crucial to estimate which of the two strands will be preferred by the ARGONAUTE (Azlan et al., 2016). Although the mechanism is still not fully understood, several constraints can be defined: This includes the presence of particular nucleotides at specific positions on the guide strand and thermodynamic parameters of the siRNA sequences. This refers to differences in the calculated minimum free energy (MFE) of the few terminal nucleotides of both ends of the siRNA chains (**Figure 1**) (Khvorova et al., 2003; Schwarz et al., 2003; Amarzguioui and Prydz, 2004; Reynolds et al., 2004; Ui-Tei et al., 2004; Frank et al., 2010; Walton et al., 2010; Noland et al., 2011;Dorsett and Tuschl, 2004). Although the thermodynamic-based parameters from both strands are expected to be consistent (since the nucleotide sequence determines the MFE), a certain discrepancy is observed. Therefore, a combination of sequence- and thermodynamic-based rules is applied in our approach in order to achieve an acceptable prediction accuracy of the siRNA efficiency (Naito et al., 2009; Walton et al., 2010; Malefyt et al., 2014).

The terminal nucleotide rule basically proposes that the presence of U or A as 5’-terminal nucleotide will predetermine this strand as a guide strand especially if it is combined with a G or C nucleotide at the 5’-end of the opposite strand. The relative thermodynamic characteristics at both ends of a siRNA double chain provide additional criteria for selection of the guide strand. The guide strand is determined as the strand with significantly lower thermodynamic stability at its 5′ terminal nucleotide in comparison with the opposite strand (**Figure 1**). This calculation was included to predict the guide strand.

### Local Accessibility of the Target Sites

Endonucleolytic cleavage of mRNA by the RISC complex requires pairing of the guide strand to the target. This interaction might be hindered, if the accessibility of the target site is covered by formation of local secondary structures (Ameres et al., 2007). To investigate the potential role of the target site accessibility for RNAi efficiency prediction, an algorithm was developed by Tafer et al. (2008). The used algorithm is based on the RNAplfold program [ViennaRNA Package 2.0, (Lorenz et al., 2011)] and computes the local base pair probabilities within an mRNA transcript for a region of length **u** within a distance **L** in a sliding window **W**. Accessibility is given in a range 0–1 (1 = very accessible, 0 = not accessible).

## Validation Experiments

To confirm the prediction performance of the new **si-Fi** algorithm, we used the *HvMlo* gene to predict the optimal region for an efficient RNAi approach. As phenotypical readout for efficient RNAi, we used the susceptibility index of barley against the mildew pathogen. In order to visualize the specificity of our software, we performed two validation experimental series.

The first approach was addressing the threshold of target specificity utilizing the “molecular clock” model. Therefore, RNAi trigger sequences were modulated and diverge from the optimal designed sequence. They were tested for their performance *in vivo*. The *HvMloH1* is used here to exemplify the unspecific off-target effect that can be expected using a related gene with more than 80% sequence identity.

The second experimental approach to validate the specificity of **si-Fi** addressed the proposed and optimized RNAi design. Here, the effect of local accessibility of target region included in the prediction of the **si-Fi** algorithm was experimentally tested. Therefore, five RNAi triggers were designed in 100-bp windows along the *HvMlo* target gene with varying sequence accessibility values.

### Test of Sequence Variation Required for RNAi Based on the “Molecular Clock” Model

In order to simulate the natural divergence that occurs between homologous genes separated either by event of speciation or gene duplication, a series of sequences with progressively increasing numbers of random mutations was generated based on the *HvMlo* sequence. The uniform rate of mutations over time is described as molecular-clock hypothesis (Margoliash, 1963; Zuckerkandl and Pauling, 1965). Based on this hypothesis, the rule of an average rate of ∼1% mutations per 1 million years was applied. This resulted in the “2% rule” (Weir and Schluter, 2008), meaning that two related genes are diverging from each other at a rate of 2% per 1 million years.

Following this rule, we have designed a set of RNAi constructs using synthetic DNA, which simulate 50 million years (MY) of evolution of the barley *Mlo* gene under neutral selection (**Table 1**), and tested them for their efficiency of silencing on *HvMlo* gene and related *HvMloH1*. The nucleotides on each altered position were selected fully randomly; therefore, it is expected that in 25% of the cases, the same nucleotide as the original is assigned. As a result, the final percent of identity is somewhat higher than the percent of the altered nucleotides (**Table 1**).

### Effect of Local Accessibility of the Target Sites

**Figure 2:**
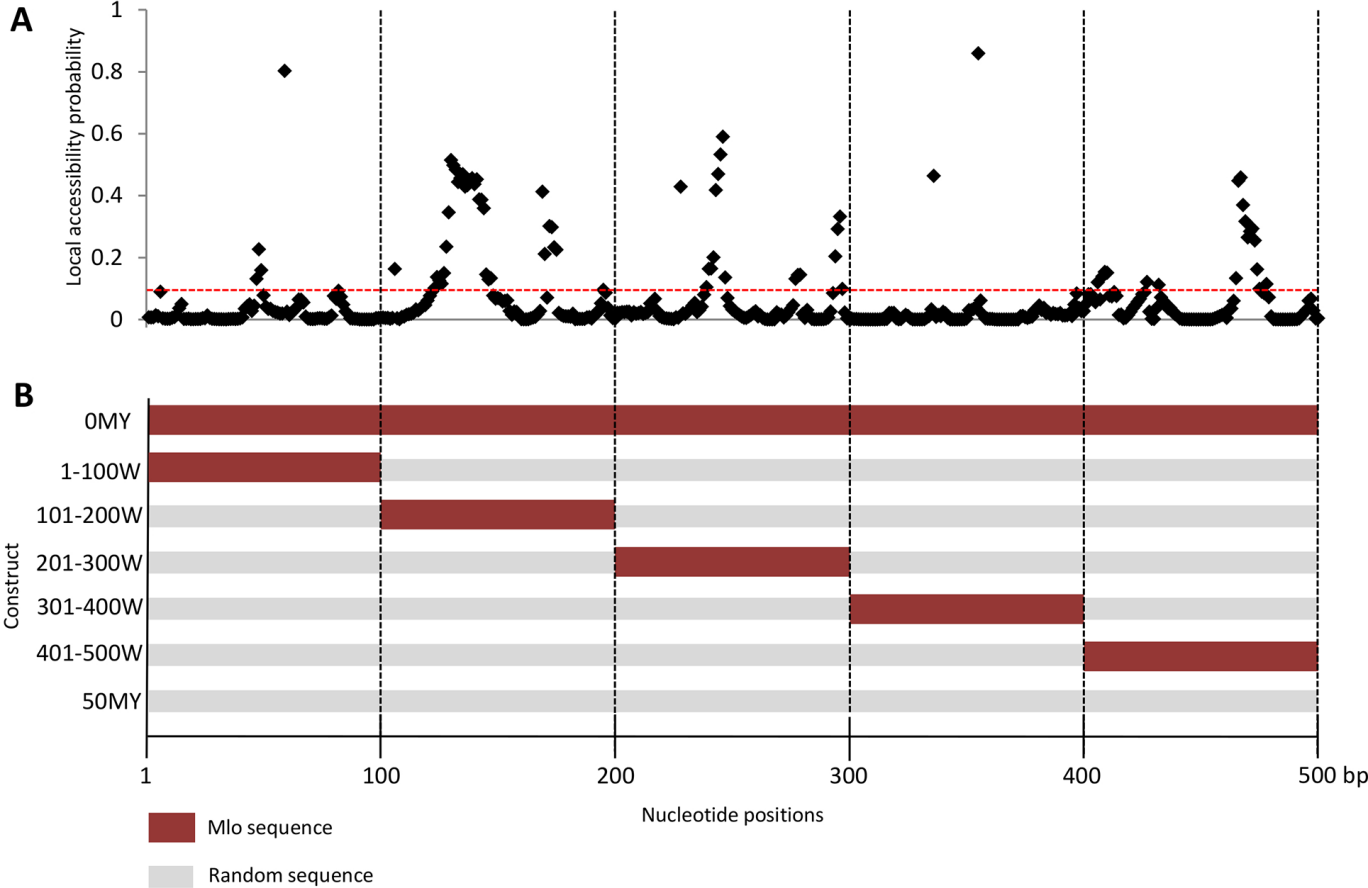
**(A)** Local accessibility probability (LAP, on the y-axis; parameters u = 8, L = 40, W = 80) of the target region of the *HvMlo* gene. Red-dotted line indicates the default **si-Fi** LAP threshold of 0.1. Please note that a high LAP is required but not sufficient condition for an efficient RNAi, and the height of the peak is probably not as decisive as long as it is higher than the threshold. **(B)** RNAi trigger sequence design of the 100-bp window constructs. The sequences of the 100-bp window constructs are derived from the 0MY (perfectly matching to the *HvMlo* target gene) and 50MY (completely randomly generated sequence) constructs as indicated by the colors (same colors stand for identical sequences in the specified segment).

As described in **Figure 2**, not all regions of the target mRNA are equally accessible to siRNAs. The local accessibility of the *Mlo* RNA target based on secondary structure fold backs was calculated by ViennaRNA Package 2.0. **Figure 2A** shows the regions of distinct properties on the example of the *HvMlo* mRNA. The coding region of *HvMlo* (500-bp sequence) was dissected into 100-bp long windows representing regions of different accessibility levels (**Figure 2B**). To compare these generated sequences with the 500-bp constructs derived from the molecular clock model, the 100-bp window constructs were filled up to 500 bp with randomized sequence originating from the 50MY construct (**Figure 2B**).

**Figure 3:**
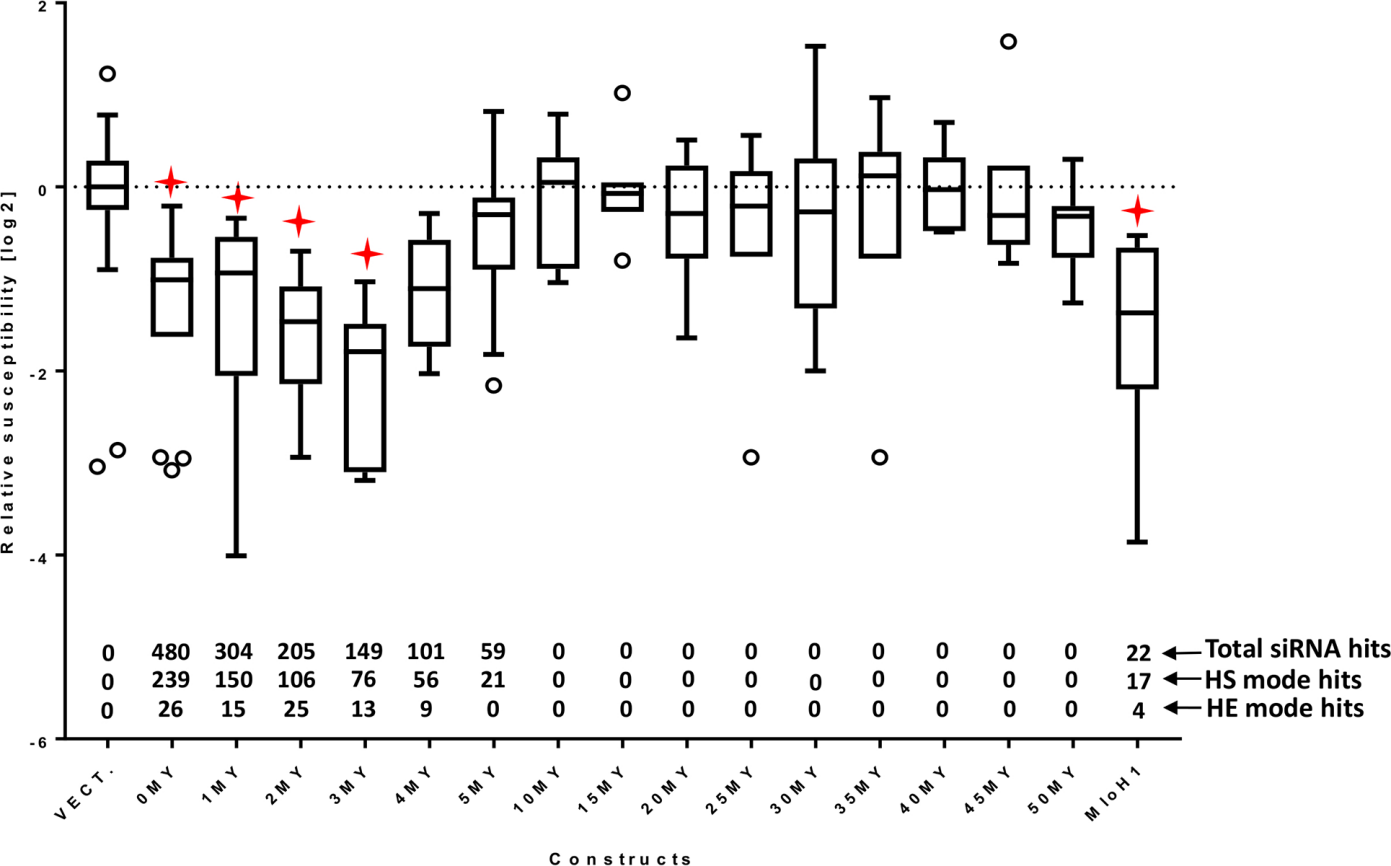
RNAi effect of the *HvMlo* and derived “molecular clock” constructs (see **Table 1**) and the *HvMloH1* construct as relative susceptibility index (RSI). The number of siRNA hits (total, high-sensitivity selection mode, and high-efficiency selection mode) is indicated above the x-axis. All RSI values are normalized to the empty vector control (VECT.) for the corresponding experiment and log_2_ transformed. Lower values indicate stronger effect (decreased susceptibility of *f.sp. hordei* against *bgh*). The boxes extend from the 25th to the 75th percentiles; the whiskers and outliers are calculated according to Tukey by GraphPad Prism (GraphPad Software, La Jolla CA) v. 7.00. Red stars indicate adjusted p-values (Dunnett’s multiple comparison test) < 0.05 in one-way ANOVA of each construct vs. empty vector control.

RNAi silencing efficiency of the generated constructs was tested in transient induced gene silencing (TIGS) experiments in detached barley leaves challenged with *Blumeria graminis f.sp. hordei (Bgh)* (Schweizer et al., 2000). Efficient silencing of the targeted *Mlo* gene is revealed by significant decrease of the susceptibility of transformed epidermal cells to *Bgh* (Humphry et al., 2006; Acevedo-Garcia et al., 2014) (**Figure 3**). Each construct was tested in a minimum of five independent biological replications (**Supplementary Table S2**). The results were normalized to the average of three parallel bombardments of the empty vector control per experiment to form a relative susceptibility index (RSI). RSI was log_2_-transformed for normal distribution of values. The 0MY construct represented the effect of an RNAi trigger that perfectly matched the target. This construct was included in every experiment as a positive control. The silencing effect of all constructs was compared against the empty vector control in a one-way ANOVA test with Dunnett’s multiple comparison correction of the p-value (**Figure 3**, **Supplementary Table S3**).

## Comparison to Previous si-Fi Versions and Alternative Software Tools

The **si-Fi** software is already a well-established tool, which has been used prior to this publication in the scientific community (Nowara et al., 2010; Lee et al., 2015; Chen et al., 2016; Wang et al., 2018). The **si-Fi** version presented is based on the previous releases, but it offers important improvements in the algorithm over the earlier **si-Fi** version:

First: The sequence-based rules for defining efficient siRNAs in the previous **si-Fi** version was replaced by a combination of thermodynamic calculation and sequence-based rules, resulting in better prediction accuracy.
Second: An additional calculation for the local accessibility of the target sites is included in the new version.
Third: The latest version presents two sets of rules, trimmed either to RNAi design or to off-target search (HE- and HS-modes, respectively).

To clearly distinguish the latest version from all previous releases, the name of the software was changed to **si-Fi21**. The results on *HvMlo*-related RNAi constructs, generated by previous **si-Fi** and the latest **si-Fi21**, are compared in **Table 2**, together with the experimentally derived mean silencing effect (increased susceptibility to powdery mildew). The results indicate that the novel version **si-Fi21** provides higher sensitivity in HS-mode (off-target prediction) and better selectivity in HE-mode (RNAi design) than the previous version. The best correlation to the experimentally derived mean silencing effect is achieved by the **si-Fi21** in HE-mode.

**Table 2:** Summary of the prediction results of the previous version of **si-Fi** and the new **si-Fi21** in bolt.

	siRNAs matching the *HvMlo* gene					
	si-Fi		si-Fi21 in bolt			
RNAi construct	Total	Efficient	Total	HS-mode	HE-mode	Mean SI
0MY*	480	90	480	26	26	-1.36
1MY*	304	51	304	150	15	-1.37
2MY*	205	53	205	106	25	-1.61
3MY*	149	44	149	76	13	-2.09
4MY	101	12	101	56	9	-1.14
5MY	59	20	59	21	0	-0.52
1–100W	80	16	80	41	0	-0.69
101–200W	80	30	80	39	7	-0.61
201–300W	81	22	81	37	7	-0.80
301–400W	80	4	80	39	1	-0.22
401–500W*	80	13	80	40	10	-1.65
MloH1*	22	0	22	17	4	-1.60
Pearson R to Mean SI	-0.31	-0.34	-0.31	-0.37	-0.61	

The predicted siRNA hits of different RNAi constructs against the Mlo gene are compared with the log^2^ transformed mean silencing effect of the constructs (mean SI) where lower SI means stronger effect. The names of construct with significant effect are marked by asterisks (*). The best correlation is achieved by the si-Fi21 in HE-mode (RNAi design mode).

**Figure 4:**
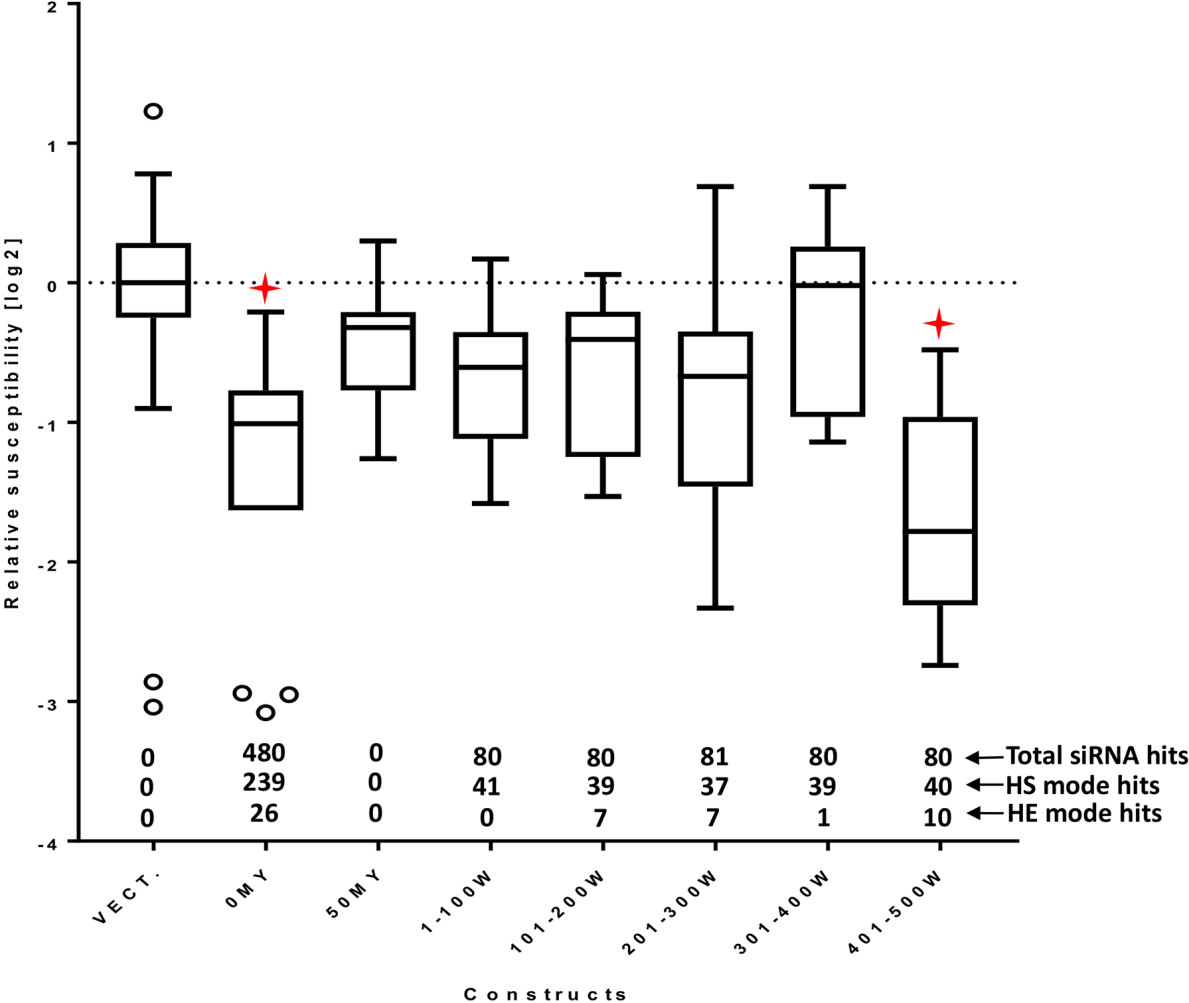
RNAi effect of the 100-bp window constructs as relative susceptibility index (RSI). The number of siRNA hits (total, high-sensitivity selection mode, and high-efficiency selection mode) is indicated above the x-axis. All RSI values are normalized to the empty vector control for the corresponding experiment and log_2_ transformed. Lower values indicate stronger effect (decreased susceptibility). The boxes extend from the 25th to the 75th percentiles; the whiskers and outliers are calculated according to Tukey by GraphPad Prism (GraphPad Software, La Jolla CA) v. 7.00. Red stars indicate adjusted p-values (Dunnett’s multiple comparison test) < 0.05 in one-way ANOVA of each construct vs. empty vector control.

Using the selected *HvMlo* reference sequence, we compared the reliability of the prediction of selected softwares: siDirect (Naito et al., 2009), dsCheck (Naito et al., 2005) and RNAi Designer (ThermoFisher, 2019), and the proposed **si-Fi21** tool (**Supplementary Table S1**). Since none of the online tools include the option to use a barley transcript database or a possibility to add a custom database, a direct comparison with the experimental results was not possible. Instead, a comparison was performed based on the number of recommended siRNAs predicted by the tools (except siDirect). The siDirect is the only one of the selected online tools able to design long double-stranded RNAi. The optimal 100-bp long sequence range for RNAi design suggested by siDirect is indicated on **Supplementary Table S1**. The design of the experiment is based on the expectation that the amount of efficient siRNAs is positively correlated with a high RNAi activity. This is in agreement with the presented example of the *HvMlo* gene, where the 401–500W construct (**Figure 4**) is triggering the strongest RNAi effect. The comparison of the RNAi tools indicates that the best fit to the experimental data is achieved by the **si-Fi21** tool in HE-mode (RNAi design). The HS-mode of **si-Fi21** (off-target search) as well as the tested online tools are in less agreement to the experimental results.

In Xu et al. (2006), the authors determined the expression levels of the *AtBTI1* gene and some of its potential off-targets in wild-type, *bti1-2* T-DNA knockdown mutant and *AtBTI1* RNAi transgenic *Arabidopsis thaliana* plants. The results indicated reduced transcript levels of the *AtBTI1* gene in the T-DNA mutant and the RNAi plants. The transcript levels of potential off-targets were investigated, and some of them were also reduced in the *AtBTI1* RNAi plants. The predictions made by the **si-Fi21** tool on the respective sequences used in this study confirm the experimentally validated off-target effects (**Table 3**). All genes with experimentally validated reduced transcript levels were successfully predicted by the HS-mode of **si-Fi21** as off-targets. A gene without predicted hits was also not affected on its transcript level by the RNAi transgene.

**Table 3:** siFi analysis results of the potential off-targets of BTI1 RNAi construct from Xu et al. (2006).

Targets	Reduced transcript levels?	Total siRNA hits	HS mode hits	HE mode hits
TC251472 (main target)	yes	808	402	41
TC251703	yes	29	14	4
TC262843	yes	3	2	0
TC269146	yes	3	2	0
TC275407	no	0	0	0

TC251472 is the BTI1 gene (the main RNAi target), the rest of the sequence potential off-targets of the BTI1 RNAi construct. The si-Fi predicted correctly all of putative off-targets with confirmed reduction of the transcript and excluded the one without transcript reduction (TC275407).

## Discussion

RNAi has become an important research tool for studying gene function. In contrast to other techniques that target the genomic DNA (e.g., CRISPR/Cas-9), RNAi offers several advantages: RNAi is able to target simultaneously multiple sequence-related transcripts. This can be, e.g., transcripts that are encoded by a gene family. The knockdown nature of RNAi also allows targeting genes, which cause lethality after knockout. Therefore, further development of tools and methods for RNAi applications remains an important task. The **si-Fi21** software tool that we present here is specifically intended for design of long double-stranded RNAi that is widely used in non-vertebrate systems. The prediction accuracy of **si-Fi21** was validated by performing multiple experiments for estimation of the gene silencing effect of specifically designed RNAi triggers. The experimental outcome was validating the results to the predicted features. Since direct measurement of the decrease of the target gene transcript is technically not feasible in this model system, we have used the phenotypic effect of the silencing of the Mlo gene as a proxy for the silencing of its transcript (Douchkov et al., 2005).

The *Mlo* silencing experiments show that the RNAi efficiency is rapidly decreasing, if the overall sequence similarity drops below 92% (4MY construct in **Figure 3**). Interestingly, the 3MY construct shows the strongest effect. However, the differences between the constructs 0MY to 3MY are not statistically significant. As the used system is based on the interaction of two living organisms, it is associated with considerable variance. This drawback can be overcome with a high number of feasible independent repetitions (**Supplementary Table S2**).

Combined analysis of the siRNA sequence and target sites allows defining siRNAs with putatively high efficiency. Applying strand selection rules (U or A at the 5’-end; MFE on the 5’-end higher than those of the 5’-end of the opposite strand; ΔMFE of the first three nucleotides of each strand >1 kcal/mol) identifies siRNAs that will be included into the RISC complex with a high probability. Calculation of the probability of local accessibility on the target (u = 8, threshold 0.1) predicts the siRNAs that are targeting accessible parts of the target RNA. These ones have a higher chance to cause a silencing effect. Combination of both selection criteria is increasing the prediction power for RNAi efficiency. However, if the criteria for selection for siRNA efficiency are set too stringent, this may cause a non-detection of some potential off-targets. Therefore, we have designed two different modes of siRNA selection. The high-sensitivity (HS) mode is using less stringent selection parameters (only strand selection rules), and it is primarily designed for finding a maximum number of potential off-targets. The high-efficiency (HE) mode is using stringent parameters (stricter strand selection rules plus target site accessibility calculations), and it will select for siRNA with strong silencing potential. The HE-mode is primarily used for RNAi construct design where maximum siRNA efficiency is desired.

The number of predicted siRNA hits in HS- and HE-modes, related to the silencing effect of the corresponding constructs, provides an estimate of **si-Fi21** prediction power (**Figure 3**). The maximum number of total, HS-, and HE-mode matching siRNAs is generated by the construct with perfect 500-bp match to the target. The 0MY construct may generate up to 480 different siRNAs with 239 and 26 of them matching the selection criteria in HS-mode and HE-mode, respectively (**Figure 3**, **Supplementary Table S4**). The number of matching siRNA is progressively decreasing parallel to the decreasing overall identity of the RNAi trigger to the target. The 1MY to 3MY constructs are predicted to generate reduced numbers of HE-mode siRNAs, whereas from the 4MY construct onward, the number of HE-mode siRNAs drops to zero. These predictions are in good agreement with the observed *Mlo* silencing effects, which were significantly weaker, compared with the 0MY reference, from 4MY onward. The results shown in **Figure 3** suggest a putative threshold mechanism, where the silencing effect is exhibited only after reaching a certain siRNA pressure on the target.

Identical experiments were performed with the 100-bp window constructs. Although all constructs are expected to generate nearly the same number of matching siRNA (80 to 81), their effect on susceptibility to *Bgh* differed substantially (**Figure 4**). Only the 401–500W construct had a significant effect on RSI. While two out of the four inefficient constructs, 101–200W and 201–300W, were associated with relatively high numbers of HE-mode siRNA predictions, they were still below the number of HE-hits of the most active 401–500W construct, which points into the direction of a threshold-dependent process of RNAi efficiency. This threshold phenomenon might be related to the short time window for observing TIGS-induced phenotypes: A suboptimal construct might not be capable of silencing the target rapidly enough to reveal the phenotypic effect, although the same construct might silence the target gradually during its continuous exposure to the siRNAs (e.g., in a transgenic plant). All 100-bp RNAi constructs gave rise to comparable numbers of HS-mode siRNAs indicating that this criterion is not sufficient to predict efficient silencing. Taken together, the results presented here suggest that the maximum number of HE-mode siRNA molecules might be a useful indicator for optimal RNAi-construct design. The identified siRNA sequences are available in **Supplementary Table S4**.

## Conclusion

**Figure 5:**
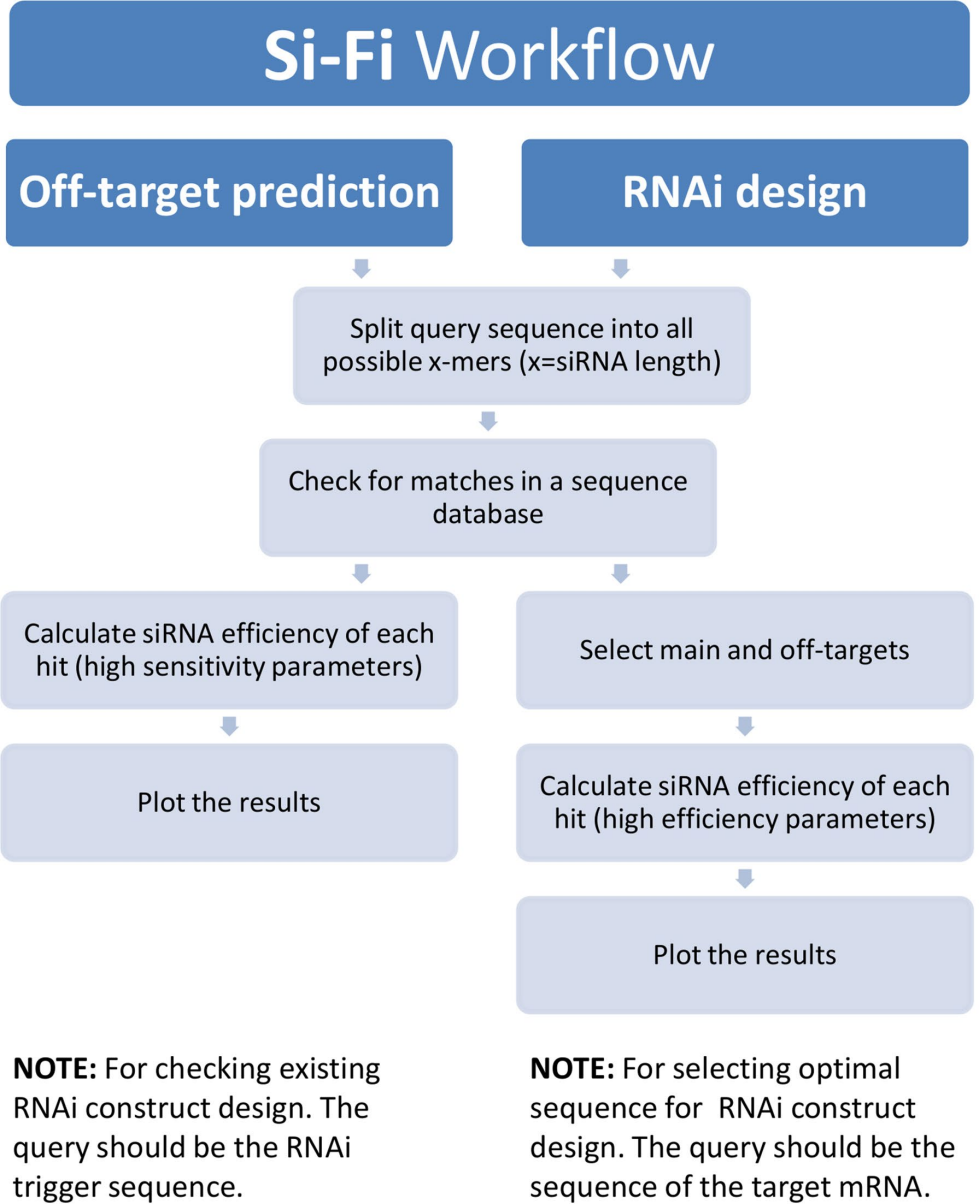
**si-Fi21** workflow diagram. The process begins with selection of the search mode. The off-target prediction is using the high-sensitivity (HS) mode settings for finding as many as possible putative off-targets as possible by minimizing the false-positive signals. The RNAi design is using the high-efficiency (HE) mode for detection of most effective siRNAs by avoiding the undesired off-targeting.

**Figure 6:**
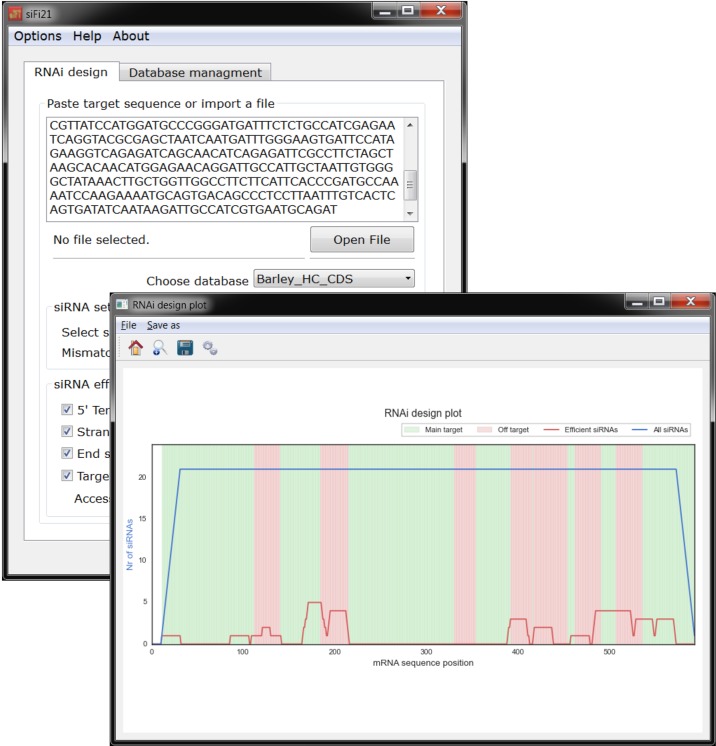
**si-Fi21** screenshots and example results of the RNAi design mode. The blue line indicates the total number of siRNA hits; the red line corresponds to the number of siRNA hits that match the selected criteria for efficiency; the red zone indicates the regions that may cause silencing of genes that were not selected as a target; in the green zone are the regions that have no match to other genes besides the selected target.

By combining the powerful BOWTIE-based sequence similarity search for putative siRNA targets together with the probability calculation of local target-site accessibility, and thermodynamics- as well as sequence-based prediction for strand selection, we have generated a Python-based software tool named si-Fi21. The software provides two different modeling modes—HS (high sensitivity) for off-target search and HE (high efficiency) for RNAi-construct design. Each of the two modes is based on an own pipeline with adapted parameters (**Figure 5**). The si-Fi21 tool is specifically intended for long double-stranded RNAi constructs including virus-, microRNA-, and host-induced gene silencing (HIGS). The software offers the possibility to create custom sequence databases, allows flexible settings of parameters, and provides easy-to-interpret graphical and tabular outputs (**Figure 6**).

## Availability and requirements

Project name: **si-Fi21**

Project home page: https://github.com/snowformatics/**siFi21**-

Operating system(s): Microsoft Windows 7 or higher

Programming language: Python

Other requirements: Python 2.7, Qt 4, PyQT4, Numpy, Matplotlib, Biopython

License: Commons Attribution-ShareAlike 4.0 International (CC BY-SA 4.0)

The MS Windows executable installation file of the version used in this article is available at Lück et al. (2017a). Newer versions will be available at Lück (2017).

## Data Availability

The datasets generated for this study can be found in e!DAL - Plant Genomics & Phenomics Research Data Repository, http://dx.doi.org/10.5447/IPK/2018/2.

## Author Contribution

SL performed research (RNAi construct preparation and testing) and programmed the software. TK performed research (RNAi construct preparation and testing). MS contributed in writing and editing the manuscript. PS designed the research and contributed in writing the manuscript. DD designed the research and the software algorithms and performed research (RNAi construct design). MK and DD wrote the manuscript. All authors read and approved the final manuscript.

## Funding

This work was funded by the Leibniz Institute of Plant Genetic and Crop Plant Research (IPK), Gatersleben, Germany.

## Conflict of Interest Statement

The authors declare that the research was conducted in the absence of any commercial or financial relationships that could be construed as a potential conflict of interest.

## Supplementary Material

Supplementary Table S1 Comparison of prediction and experimentaly validated regions for RNAi efficience

Supplementary Table S2 Experimental results of the TIGS experiments. In total 15 independent experiments were conducted, where each of the construct was tested in at least 5 of them. The values indicate the Relatively Susceptibility Index (RSI) of each construct calculated as follows: Each experiment includes three empty vector controls. The mean of the three values was set to 100% susceptibility and the results of the rest of the constructs of the same experiment were calculated accordingly as percent to the mean of the empty vector controls thus forming the RSI.

Supplementary Table S3 Statistical summary of the Dunnet’s multiple comparison test of control (empty vector) vs. test constructs. VECT = empty vector control.

Supplementary Table S4 Summary of all putative siRNAs with their predicted properties. The columns content is as follows:“siRNA_sequence” – sequence of the siRNA; “siRNA_name” – programm internal sequence identifier; “siRNA_position” – start position of the siRNA based on the trigger sequence ruler; “HS_mode_selected” – whether or not the siRNA meets the criteria of the HS-mode selection; “HE_mode_selected” - whether or not the siRNA meets the criteria of the HE-mode selection; “hit_name” – sequence identifier of the matched target; “mismatches” – number of mismatches to the target sequence; “is_off_target” – whether the match is to the main target or to a off-target based on the user selection; “accessibility_value” – target site local accessibility value; “target_site_accessibility_>0.1” – indicated whether or not the target site local accessibility is higher that the selected threshold (in this case 0.1); “reference_strand_pos” – start position of the siRNA target sequence based on the target sequence ruller; “end_stability_positive” – indicates whether or not the sequence meet the criteria for end-stability rules; “strand_selection_positive” – indicates whether or not the sequence meets the criteria for strand selection.
