# Supplementary material for: siRNA-Finder (si-Fi) Software for RNAi-Target Design and Off-Target Prediction
Source: Front Plant Sci. 2019 Aug 15;10:1023. doi: 10.3389/fpls.2019.01023 (PMC6704232; doi:10.3389/fpls.2019.01023)
Supplement: Supplementary file 5 [file Presentation_1.pptx]

## Slide 1
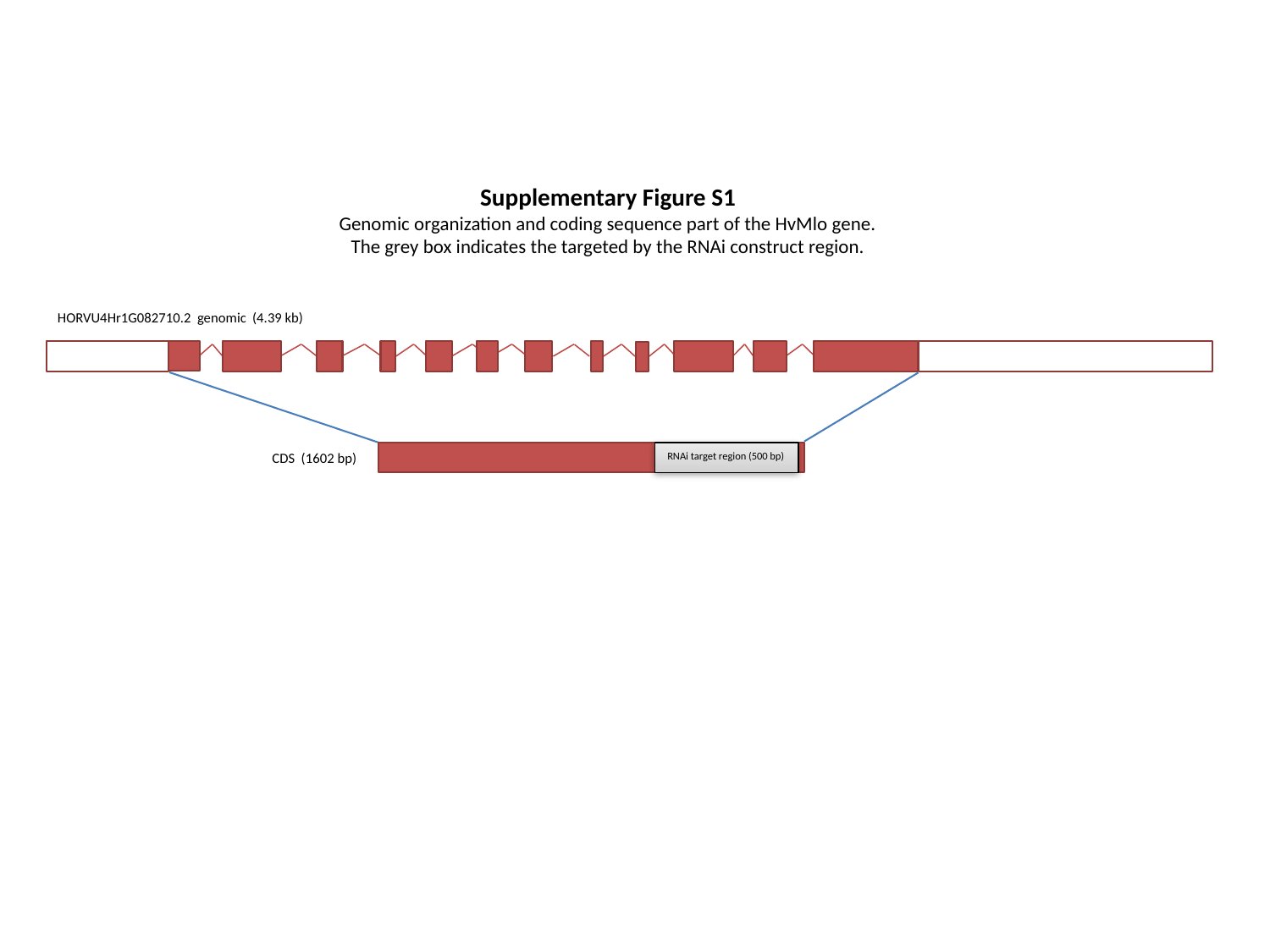

Supplementary Figure S1
Genomic organization and coding sequence part of the HvMlo gene.
The grey box indicates the targeted by the RNAi construct region.
HORVU4Hr1G082710.2 genomic (4.39 kb)
CDS (1602 bp)
RNAi target region (500 bp)
